# Supplementary material for: Content-rich biological network constructed by mining PubMed abstracts
Source: BMC Bioinformatics. 2004 Oct 8;5:147. doi: 10.1186/1471-2105-5-147 (PMC528731; doi:10.1186/1471-2105-5-147)
Supplement: Additional File 2 — The original results of the above study (non-essential files are deleted to keep the file size under the limit set by BMC bioinformatics). [file 1471-2105-5-147-S2.bz2 › chilibotAdditionalFile2/dip20/296ID7486683E1287/html/GRB2_IRS1.html]

 


 **GRB2** and **IRS1** 
  
Found 180 abstracts in PubMed, retrieved 20.  
 

 What does Google say? 
 PDF only 
| .edu only 

---

**Interactive relationship** (e.g. stimulation, inhibition, etc)

**Inhibitory relationship**- With regard to the large effect on MAPK phosphorylation, we found that PTP1B overexpression led to the impaired phosphorylation of both IRS 1  [ **IRS1** ]  and Shc, resulting in a decrease in their association with  **Grb2** .  Ref: 12446583 Endocrinology, 2002
**Stimulatory relationship**- Moreover, the GH induced association of IRS 1  [ **IRS1** ]  phosphatidylinositol 3 kinase, IRS 1  [ **IRS1** ]  growth factor receptor bound 2  **Grb2** , and Shc  **Grb2**  are increased in diabetic rats as well.  Ref: 12086960 Diabetes, 2002
- Although phosphatidylinositol 3 kinase PI3K and Grb 2  [ **GRB2** ]  interact with tyrosine phosphorylated IRS  [ **IRS1** ]  proteins, Shp 2 only binds to IRS  [ **IRS1** ]  proteins following IL 4, but not IL 9, stimulation.  Ref: 11788580 J Biol Chem, 2002
**Neutral relationship**- Signalling by the insulin receptor substrate IRS  [ **IRS1** ]  proteins is critically dependent on the tyrosine phosphorylation of specific binding sites that recruit Src homology 2 SH2 domain containing proteins, such as the p85 subunit of phosphoinositide 3 kinase PI 3 kinase, the tyrosine phosphatase SHP 2 and the adapter protein  **Grb2** .  Ref: 12220227 Biochem J, 2002

**Non-interactive relationship** (e.g. studied together, co-existance, homology, etc.)

- Data presented here provide strong evidence that IGF IR deficient brown adipocytes show an increased insulin sensitivity via IRS 1  [ **IRS1** ]  Grb 2  [ **GRB2** ]  MAPK, resulting in an increased mitogenesis in response to insulin.  Ref: 11872675 Diabetes, 2002
- The nanomolar affinity of the mAZ pY alphaMe pY N NH2 pseudo peptide for  **Grb2**  SH2 is related to new interactions with non conserved residues.The design of  **Grb2**  SH2 domain inhibitors that prevent interaction with tyrosine kinase proteins or other adaptors like Shc or  **IRS1**  should provide a means to interrupt the Ras signaling pathway.  Ref: 11827484 J Mol Biol, 2002
- We show that in Drosophila mutating the consensus binding site for the Ras pathway adaptor Drk  **Grb2**  in Chico IRS  [ **IRS1** ]  does not interfere with growth whereas mutating the binding sites of the PI 3 kinase adaptor p60 completely abrogates Chico function.  Ref: 12163412 Development, 2002
- Downstream, the association IRS 1  [ **IRS1** ]  growth factor receptor binding protein 2 Grb 2  [ **GRB2** ]  was augmented in the IGF IR brown adipocyte cell line.  Ref: 11872675 Diabetes, 2002
- The results from the overexpression of IRAS, where its amount was about the same as that of IRS  [ **IRS1** ]  4, indicated that IRAS associated directly with IRS  [ **IRS1** ]  4 and showed that the increased complexation of IRS  [ **IRS1** ]  4 with IRAS did not alter the insulin stimulated tyrosine phosphorylation of IRS  [ **IRS1** ]  4 or the association of IRS  [ **IRS1** ]  4 with phosphatidylinositol 3 kinase or  **Grb2** .  Ref: 11912194 J Biol Chem, 2002
- IRS 1  [ **IRS1** ]  association with  **Grb2**  and PI3K p85, which link IGFRI to the Ras MAPK and PI3K Akt pathways, respectively, was enhanced in both cell types in the presence of EN.  Ref: 12173038 Oncogene, 2002
- In response to IGF 1 treatment, the expression of kinases in the c Jun c Jun NH 2 terminal kinase signaling pathway MEKK1, MEK4, and JNK1 2 was unchanged, but expressions of insulin receptor substrate 1  [ **IRS1** ]  and  **Grb2**  associated binder 1 Gab1 were significantly decreased.  Ref: 12065326 Circ Res, 2002
- EGF increased the tyrosine phosphorylation of several proteins the EGF receptor, insulin receptor substrate IRS 1  [ **IRS1** ] , IRS  [ **IRS1** ]  2, and  **Grb2**  associated binder 1.  Ref: 12138086 J Biol Chem, 2002
- TGF beta1 affected IGF I stimulated insulin receptor substrate 1  [ **IRS1** ]  IRS 1  [ **IRS1** ]  tyrosine phosphorylation and its association with  **Grb2**  protein.  Ref: 12837295 Exp Cell Res, 2003
- Treatment with 17 AAG decreased NPM ALK expression and phosphorylation, thus impairing its association with phospholipase C gamma, Src homology 2 domain containing protein Shc, growth factor receptor bound protein 2  [ **GRB2** ]   **Grb2** , and insulin receptor substrate 1  [ **IRS1** ]  IRS 1  [ **IRS1** ] .  Ref: 11888936 Cancer Res, 2002
- Conversely, TGF beta1 did not alter IGF I stimulated IRS 1  [ **IRS1** ]  associated phosphatidylinositol 3 kinase activity, IGF I induced tyrosine phosphorylation of Shc, and its binding to  **Grb2** .  Ref: 12837295 Exp Cell Res, 2003
- We observed association between BCR ABL IRS 1  [ **IRS1** ] , IRS 1  [ **IRS1** ]  phosphoinositide 3 kinase PI3 kinase, and IRS 1  [ **IRS1** ]   **Grb2**  in the K562 cell line.  Ref: 12560071 FEBS Lett, 2003
